# Plasma ceramides containing saturated fatty acids are associated with risk of type 2 diabetes

**DOI:** 10.1016/j.jlr.2021.100119

**Published:** 2021-09-20

**Authors:** Amanda M. Fretts, Paul N. Jensen, Andrew N. Hoofnagle, Barbara McKnight, Barbara V. Howard, Jason Umans, Colleen M. Sitlani, David S. Siscovick, Irena B. King, Luc Djousse, Nona Sotoodehnia, Rozenn N. Lemaitre

**Affiliations:** 1 Department of Epidemiology, University of Washington, Seattle, WA, USA; 2 Cardiovascular Health Research Unit, University of Washington, Seattle, WA, USA; 3 Department of Medicine, University of Washington, Seattle, WA, USA; 4 Department of Laboratory Medicine, University of Washington, Seattle, WA, USA; 5 Department of Biostatistics, University of Washington, Seattle, WA, USA; 6 MedStar Health Research Institute, University of Washington, Hyattsville, MD, USA; 7 Center for Clinical and Translational Science, Georgetown and Howard Universities, Washington, DC, USA; 8 New York Academy of Medicine, New York, NY, USA; 9 Department of Internal Medicine, University of New Mexico, Albuquerque, NM, USA; 10 Department of Medicine, Brigham and Women's Hospital and Harvard Medical School, Boston, MA, USA

**Keywords:** epidemiology, elderly, ceramides, sphingomyelins, diabetes, cohort study, cardiovascular disease, hazard ratios, Cox proportional hazards, saturated fatty acids, Cer, ceramide, CHD, coronary heart disease, CHS, Cardiovascular Health Study, CRP, C-reactive protein, CVD, cardiovascular disease, NHLBI, National Heart, Lung, and Blood Institute

## Abstract

Recent studies suggest that the type of saturated fatty acid bound to sphingolipids influences the biological activity of those sphingolipids. However, it is unknown whether associations of sphingolipids with diabetes may differ by the identity of bound lipid species. Here, we investigated associations of 15 ceramide (Cer) and SM species (i.e., all sphingolipids, measured with coefficient of variation less than 20%) with incident type 2 diabetes in the Cardiovascular Health Study (n = 3,645), a large cohort study of cardiovascular disease among elderly adults who were followed from 1989 to 2015. Diabetes incidence was defined as fasting glucose ≥126 mg/dl or nonfasting glucose ≥200 mg/dl; reported use of insulin or oral hypoglycemic medication; or documentation of diabetes diagnosis through the Centers for Medicare and Medicaid Services records. Associations of each sphingolipid with incident diabetes were assessed using a Cox proportional hazards regression model. We found that higher circulating levels of Cer with acylated palmitic acid (Cer-16), stearic acid containing Cer (Cer-18), arachidic acid containing Cer (Cer-20), and behenic acid containing Cer (Cer-22) were each associated with a higher risk of diabetes. The hazard ratios for incident diabetes per 1 SD higher log levels of each Cer species were as follows: 1.21 (95% CI: 1.09–1.34) for Cer-16, 1.23 (95% CI: 1.10–1.37) for Cer-18, 1.14 (95% CI: 1.02–1.26) for Cer-20, and 1.18 (95% CI: 1.06–1.32) for Cer-22. In conclusion, higher levels of Cer-16, Cer-18, Cer-20, and Cer-22 were associated with a higher risk of diabetes.

Type 2 diabetes is a major risk factor for cardiovascular disease (CVD). Although many risk factors for diabetes are well established (e.g., obesity, poor diet, physical inactivity), the burden of diabetes among older adults remains high. Identifying potentially modifiable biological mechanisms that influence the development of diabetes is of public health importance.

Sphingolipids are a class of lipids containing a backbone sphingoid base (sphingosine) and one N-acylated fatty acid. The acylated fatty acid of sphingolipids is often saturated and varies in number of carbon atoms. Sphingosine N-acylated with a fatty acid forms ceramides (Cers), and different head groups attach to Cers to form more complex sphingolipids, such as phosphorylcholine to form sphingomyelins (1). In plasma, sphingolipids comprise a part of lipoproteins (HDL, LDL, and very LDL) (2).

Previous work in cells and animals provides evidence that sphingolipids influence diabetes-related phenotypes. High levels of circulating Cers and sphingomyelins have consistently been shown to be associated with impaired insulin signaling and glucose transport, insulin resistance, inflammation, oxidative stress, and beta-cell apoptosis (3, 4, 5, 6, 7, 8, 9, 10). In addition, animal studies indicate that insulin sensitivity and glucose homeostasis are improved by inhibition of de novo sphingolipid synthesis (8, 9, 11, 12, 13).

Recent studies in humans suggest that the type of saturated fatty acid bound to Cers influences the biological activity of Cers, and that the magnitude of associations of sphingolipids with incident diabetes may differ by lipid species (14, 15, 16, 17, 18, 19). For instance, results from a meta-analysis that we performed among American Indian adults in the Strong Heart Study and Strong Heart Family Study suggest that higher levels of stearic acid containing Cer (Cer-18), arachidic acid containing Cer (Cer-20), and behenic acid containing Cer (Cer-22), but not Cer with acylated palmitic acid (Cer-16), or lignoceric acid containing Cer (Cer-24), are associated with a higher risk of developing diabetes (15). In the Strong Heart Study and Strong Heart Family Study, we did not observe associations of other measured sphingolipids (i.e., sphingomyelins, glucosyl-Cers, or lactosyl-Cers) with diabetes risk (15). To date, most published studies that have examined associations of Cer and SM species with diabetes risk focus primarily on young or middle-aged populations, and little is known about the associations of sphingolipid species with risk of diabetes in the elderly. As diabetes is a heterogeneous disorder, and diabetes that develops among the elderly can be metabolically distinct from younger-onset diabetes (20), and circulating levels of some sphingolipid species have been shown to vary according to age (21), better understanding the association of sphingolipid species with diabetes risk in the elderly is needed.

The purpose of this article was to examine the associations of sphingolipid species with a saturated fatty acid acylated to the sphingoid backbone with the risk of incident type 2 diabetes among older adults (aged 65 years or older at enrollment) who participated in the Cardiovascular Health Study (CHS), a large community-based prospective cohort study. In total, concentrations of 22 different saturated fatty acids containing sphingolipid species were measured using liquid chromatography-tandem mass spectrometry. This analysis focuses on the 15 Cer and SM species measured with analytical coefficient of variation less than 20%.

## Materials and methods

### Design and population

The CHS is a prospective community-based cohort study of CVD among adults 65 years of age or older from four communities in the United States (Forsyth County, North Carolina; Sacramento County, California; Washington County, Maryland; and Allegheny County, Pennsylvania). The study design and sampling methods have been previously described in detail (22). Medicare eligibility lists were used to randomly select noninstitutionalized adults aged 65 years or older from the participating communities. In 1989–1990, 5,201 participants enrolled in the study, and in 1992–1993, an additional 687 participants (predominantly black) enrolled. Participants were followed by annual clinic visits with interim phone calls for the first 10 years of the study and then by phone contact two times per year thereafter. Participants also completed an in-person clinic visit in 2005–2006. The University of Washington institutional review board approved the study, and written informed consent was obtained from study participants at enrollment. This study abides by the principles of the Declaration of Helsinki.

There were 22 plasma sphingolipid species measured on 4,026 participants using specimens from the 1994–1995 clinic visit that had been stored at −70 °C. In addition, among 586 participants without available plasma specimens from the 1994–1995 clinic visit, specimens from the 1992–1993 clinic visit were used to measure the sphingolipid species. After excluding participants: with prevalent diabetes at the time of the sphingolipid measurement (n = 898); with unknown diabetes status at the time of the sphingolipid measurement (n = 3); and who did not participate in follow-up (n = 66), 3,645 participants were included in the analytic cohort for this report.

### Data collection

Standardized interviews, physical examinations, laboratory evaluations, and diagnostic testing were performed at the annual clinic visits (22, 23). Blood samples were collected after a 12-h overnight fast and stored at −70 °C. Fasting glucose was measured at the clinic visits in 1992–1993, 1996–1997, 1998–1999, and 2005–2006; and nonfasting glucose was measured at the 1994–1995 clinic visit. Medication use was assessed annually through the first 10 years of the study and again in 2005–2006 by an in-person inventory of medications used during the past 2 weeks at the clinic visits. During years without clinic visits (2000–present; except 2006–2006 in which there was an in-person clinic visit), medication use was assessed via semiannual phone call. BMI, waist circumference, and blood pressure were assessed using standardized methods, as reported previously (22, 23). Standardized questionnaires were administered at the clinic exams to collect information on education, medical history, smoking, alcohol consumption, and physical activity (22).

### Sphingolipid measurement

As we were most interested in sphingolipids with a saturated fatty acid acylated to the sphingoid backbone, we measured 22 saturated fatty acids containing sphingolipids using stored samples from 1992–1993 or 1994–1995. For the current investigation, we restricted analyses to the 15 sphingolipid species with coefficients of variation less than or equal to 20%. This included five Cer species (i.e., Cer-16, Cer-18, Cer-20, Cer-22, and a composite concentration of Cer-24, computed as the sum of the concentrations of lignoceric acid containing two species of Cers with the distinct “d18:1” and “d18:2” sphingoid backbones); six SM species (i.e., myristic acid containing SM [SM-14], palmitic acid containing SM [SM-16], stearic acid containing SM [SM-18], arachidic acid containing SM [SM-20], behenic acid containing SM [SM-22], and lignoceric acid containing SM [SM-24]); 3 hexosyl-Cers (i.e., palmitic acid containing hexosyl-Cer [HexCer-16], behenic acid containing hexosyl-Cer [HexCer-22], and lignoceric acid containing hexosyl-Cer [HexCer-24]); and one lactosyl-Cer (i.e., palmitic acid containing lactosyl-Cer [LacCer-16]). The sphingoid backbone for all sphingolipids measured other than Cer-24 was d18:1 (24). Plasma lipids were extracted, and sphingolipids were quantified by liquid chromatography-tandem mass spectrometry. Notably, glucosyl-Cer and lactosyl-Cer are not resolved by this chromatographic program. As a result, we use the term hexosyl-Cer to refer to the signal from these isomeric lipids. Concentrations of sphingolipid species (expressed as micrometer) were quantified using a single point calibrator made from a pooled EDTA plasma sample and added to each batch in five replicates. Concentrations of HexCer-16, HexCer-22, HexCer-24, and LacCer-16 were based on internal standards because external standards were not available at the time to spike into the single-point calibrator; these concentrations were adjusted to account for the difference in recovery of internal versus external standards for LacCer-24 (25). The detailed methodology and quality control procedures have been published (24, 26). All sphingolipids were measured by the Analytic Core of the Nutrition Obesity Research Center at the University of Washington (Seattle, WA).

### Ascertainment of diabetes

Incident diabetes was defined using information collected as part of the clinical examinations as well as information available through the Centers for Medicare and Medicaid Services records. Participants were considered to have diabetes if: glucose ≥126 mg/dl when participants reported fasting ≥8 h before venipuncture; or glucose ≥200 mg/dl when fasting was <8 h; or reported use of insulin or oral hypoglycemic medication; or Centers for Medicare and Medicaid Services records show two or more inpatient (i.e., hospital, nursing home, or home health services), three or more outpatient (outpatient or carrier health services), or at least one inpatient and one outpatient International Classification of Diseases, Ninth Revision, Clinical Modification Medicare claim codes for diabetes diagnosis (i.e., prefix 250.xx at any position within the claim) over a 2-year period.

### Calculation

All statistical analyses were conducted using STATA, version 14.0 (Stata Corp, College Station, TX). To describe the baseline demographic, lifestyle, and clinical characteristics of study participants according to the sphingolipid species of interest, each sphingolipid species was categorized into quartiles. Cox proportional hazards regression was used to examine associations of circulating sphingolipid species levels with incident diabetes, with entry at the time of the sphingolipid measurement and time at risk until first diagnosis, death, or the latest adjudicated date of follow-up. Sphingolipid species concentrations were log transformed because of skewness. Observed associations were quantified using hazard ratios for diabetes per one SD in log sphingolipid species concentration (micromolar). A Bonferroni correction was used to adjust for multiple comparisons; the significance threshold of 0.0033 (0.05/15 sphingolipid species) was used. Schoenfeld residual tests were used to evaluate the proportional hazards assumption for each sphingolipid of interest.

Two levels of adjustment were used to examine associations of sphingolipids with incident diabetes. The first model (primary model) adjusted for a priori confounders, including age, sex, race (white or black), enrollment site (Bowman Gray, Davis, Hopkins, and Pittsburg), education (no high school, high school/vocational school, and college), year of sphingolipid measurement (1992–1993 or 1994–1995), smoking (never, former, and current), physical activity (blocks walked per week), BMI (kg/m^2^), waist circumference (cm), and LDL cholesterol (mg/dl) at the time of the sphingolipid measure. In a second model (exploratory model), we in addition adjusted for prevalent coronary heart disease (CHD) (yes/no) at the time of the sphingolipid measure to assess if CHD mediates or confounds the relationship of each sphingolipid of interest with incident diabetes. Martingale residuals plotted against the continuous covariate values with a lowess smoother were used to assess the functional form of key covariates (i.e., sphingolipids, age, education, BMI, waist circumference, physical activity, and LDL cholesterol); as no departure from linearity was observed, these variables were modeled linearly. Missing values of LDL (n = 175), BMI (n = 6), smoking (n = 4), physical activity (n = 41), and waist circumference (n = 78) were imputed by chained equations using information on age, sex, race, and weight. Twenty imputed datasets were generated, and model fitting results were pooled using standard methods (26).

We conducted a number of sensitivity analyses. To better understand whether markers of kidney function or inflammation influence observed associations, we evaluated models that further adjusted for creatinine and C-reactive protein (CRP) levels at the time of the sphingolipid measure. We also ran analyses that further adjusted for alcohol use, HDL cholesterol, triglycerides, and systolic blood pressure to better understand if these factors influenced observed associations. As several of the Cer species of interest are correlated, we evaluated models that adjusted for one of the other species to potentially distinguish the associations of Cers with long chain saturated fatty acids from Cers with very long saturated fatty acids, as has been previously done elsewhere (27, 28). The Cer-16 model included adjustment for Cer-22, whereas the Cer-20, Cer-22, and Cer-24 models included adjustment for Cer-16.

As the associations of sphingolipids with diabetes risk may differ by age or cardiovascular health status, we examined the potential interaction of each sphingolipid of interest with age, sex, CHD (yes/no), and BMI at the time of the sphingolipid measure to investigate whether these factors modify the association of sphingolipids with diabetes. Likelihood ratio tests were used to evaluate the statistical significance of the multiplicative interaction term for each factor with each sphingolipid of interest modeled linearly.

## Results

Among the 3,645 CHS participants in the analytic cohort, 61% were female, and the mean (SD) age at the time of the sphingolipid measurement was 77 (5) years. Mean (SD) BMI was 26.2 (4.5) kg/m^2^, and 21% had prevalent CHD at the time of the sphingolipid measurement. The distribution of circulating levels of sphingolipid species is shown in Table 1.

**Table 1:** Concentrations (micromolar) of plasma sphingolipid species containing acylated saturated fatty acids

Sphingolipid	Mean ± SD	Range
Cer-16	0.26 ± 0.06	0.12–0.80
Cer-18	0.18 ± 0.07	0.04–0.64
Cer-20	0.08 ± 0.03	0.01–0.24
Cer-22	0.61 ± 0.18	0.18–1.90
Cer-24	4.42 ± 1.05	1.55–9.98
SM-14	36.5 ± 0.70	5.76–95.7
SM-16	125.9 ± 19.0	48.9–208.8
SM-18	37.9 ± 9.70	12.8–129.2
SM-20	17.8 ± 3.52	6.19–35.9
SM-22	26.6 ± 5.78	9.60–63.2
SM-24	14.3 ± 3.49	4.59–33.2
HexCer-16^a^	2.05 ± 0.76	0.54–6.80
HexCer-22^a^	1.90 ± 0.65	0.76–5.61
HexCer-24^a^	1.94 ± 0.54	0.54–5.40
LacCer-16^a^	7.99 ± 2.38	2.05–21.9

a Concentrations corrected for differences in recovery of internal versus external standards for these species.

**Fig. 1:**
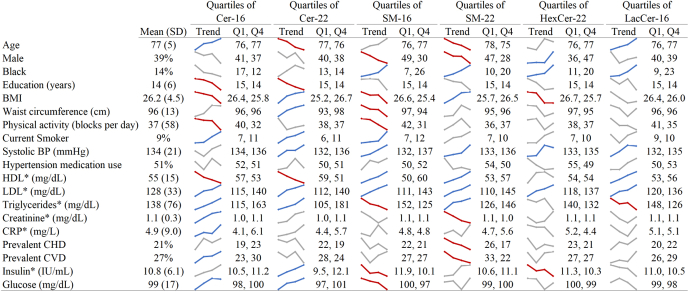
Baseline characteristics of study participants according to quartiles of select sphingolipid species of interest. ∗ denotes variables only measured at the exam in 1992–1993. The value of each covariate for the first and last quartile is provided in the figure (Q1 and Q4). The values of each covariate across the four quartiles are also depicted with spark lines. The colors of the spark lines denote statistical significance of a test for trend based on a Bonferroni-corrected *P* value of 0.05/19 = 0.0026 (based on 19 baseline characteristics of interest): Red, inverse association; gray, no association; blue, positive association. BP, blood pressure; Cer-16 and Cer-22, ceramides with palmitic and behenic acid, respectively; HexCer-22, hexosylceramide with behenic acid; LacCer-16, lactosylceramide with palmitic acid; SM-16 and SM-22, sphingomyelins with palmitic and behenic acid, respectively.

Demographic and cardiometabolic characteristics of the study participants at the time of the sphingolipid measurement according to quartile of select sphingolipid species are shown in Fig. 1 and supplemental Figs. S1–S3. Although there were several observed associations of the characteristics with the sphingolipid species, the direction of the associations differed across individual sphingolipid species, with some species showing divergent associations for Cer and SM species. For instance, higher levels of systolic blood pressure and LDL cholesterol were associated with higher levels of most Cer and SM species. On the other hand, higher levels of HDL cholesterol were associated with lower levels of Cer-16 and Cer-22, and higher levels of SM-16, SM-22, and LC-16, whereas higher levels of glucose and triglycerides were associated with higher levels of Cer-16 and Cer-22 but lower levels of SM-16.

During 33,462 person-years of follow-up (mean follow-up: 9.2 years), 434 participants developed diabetes. Higher levels of circulating Cer-16, Cer-18, Cer-20, and Cer-22 were each associated with a higher risk of diabetes after adjustment for age, sex, race, enrollment site, education, smoking, physical activity, BMI, waist circumference, LDL cholesterol, and year of sphingolipid measurement (1992–1993 or 1994–1995) (Table 2). Cer-24, all SM species (i.e., SM-14, SM-16, SM-18, SM-20, SM-22, SM-24), hexosyl Cers (i.e., HexCer-16, HexCer-22, HexCer-24), and the lactosyl-Cer (i.e., LacCer-16) were not associated with diabetes risk.

**Table 2:** HRs (95% CI) for incident diabetes for plasma sphingolipids with saturated fatty acids

	Model 1		Model 2	
Sphingolipid species	Hazard Ratio (95% CI)	*P*	Hazard Ratio (95% CI)	*P*
Cer-16	1.21 (1.09, 1.34)	4.0 × 10^−4^	1.21 (1.09, 1.34)	5.0 × 10^−4^
Cer-18	1.23 (1.10, 1.37)	2.0 × 10^−4^	1.22 (1.10, 1.36)	3.0 × 10^−4^
Cer-20	1.14 (1.02, 1.26)	2.1 × 10^−3^	1.13 (1.01, 1.26)	2.9 × 10^−3^
Cer-22	1.18 (1.06, 1.32)	2.0 × 10^−3^	1.19 (1.06, 1.32)	2.0 × 10^−3^
Cer-24	1.10 (0.99, 1.23)	0.08	1.10 (0.99, 1.23)	0.08
SM-14	0.90 (0.80, 1.00)	0.06	0.90 (0.81, 1.01)	0.07
SM-16	0.93 (0.83, 1.04)	0.22	0.93 (0.83, 1.04)	0.22
SM-18	1.03 (0.91, 1.15)	0.67	1.02 (0.91, 1.15)	0.71
SM-20	0.97 (0.87, 1.08)	0.55	0.97 (0.88, 1.08)	0.62
SM-22	1.05 (0.94, 1.17)	0.42	1.05 (0.94, 1.18)	0.37
SM-24	1.02 (0.91, 1.14)	0.71	1.03 (0.92, 1.15)	0.64
HexCer-16	0.99 (0.89, 1.11)	0.90	0.99 (0.89, 1.10)	0.82
HexCer-22	0.93 (0.84, 1.03)	0.18	0.93 (0.84, 1.03)	0.18
HexCer-24	0.90 (0.81, 1.00)	0.06	0.90 (0.81, 1.00)	0.05
LacCer-16	0.93 (0.84, 1.03)	0.17	0.93 (0.84, 1.03)	0.16

HRs (95% CI) for diabetes per one SD in log sphingolipid species concentration (micromolar).

Model 1 adjusted for age, sex, race, enrollment site, education, smoking, physical activity, BMI, waist circumference, LDL cholesterol, and year of sphingolipid measurement. Model 2 in addition adjusted for prevalent coronary heart disease.

There were no statistically significant interactions between the sphingolipid species and age (modeled linearly), sex, CHD (yes/no), or BMI when assessing risk of diabetes (smallest *P* for interaction = 0.05). Sensitivity analyses that further adjusted for creatinine and CRP levels or for alcohol use, HDL cholesterol, and systolic blood pressure produced similar results (data not shown). However, additional adjustment for triglycerides attenuated associations for Cer-20 and Cer-22 but not Cer-16 and Cer-18 with diabetes risk (supplemental Table S1).

## Discussion

In this large community-based prospective study of older adults, circulating levels of Cer-16, Cer-18, Cer-20, and Cer-22 were each positively associated with diabetes risk. On the other hand, circulating sphingomyelins, hexosyl-Cers, or lactosyl-Cers were not associated with diabetes risk. These findings suggest that among the 15 sphingolipids included in this analysis (i.e., all the sphingolipids measured with a coefficient of variation less than 20%), only Cers are associated with the risk of diabetes independent of the length of their acylated saturated fatty acid.

Our results are similar to findings from previous studies that suggest that plasma or serum Cers are positively associated with risk of diabetes, and that associations are generally similar across Cer species (14, 15, 17). In particular, our findings are strikingly consistent with our previous work in the Strong Heart Study and Strong Heart Family Study (15). In the Strong Heart Study and Strong Heart Family Study, American Indians who were 14+ years old (mean age in the Strong Heart Study and Strong Heart Family Study was 57 and 37 years, respectively) had 18–22% higher risk of developing diabetes per one SD of each log Cer species (i.e., Cer-18, Cer-20, Cer-22) (15). Our results are also consistent with findings from the Latino Health Study (mean ages: 34–41 years), which showed that serum Cer-16 and Cer-18 are associated with a higher risk of diabetes (17); and the FINRISK 2002 Study (mean age: 44 years), which showed that Cer-18 and Cer-24 are positively associated with incident diabetes (14). Our results contradict findings from an untargeted lipidomics analysis in Chinese Singaporeans (median age: 47 years) that reported no associations of Cer-16, Cer-18, Cer-20, Cer-22, or Cer-24 with diabetes risk (16). Making sense of this inconsistent finding is challenging and may be due to differences in the underlying study population (i.e., race/ethnicity, health behaviors or lifestyle factors, underlying clinical risk factors) or the different lipidomic approaches used to measure sphingolipids. Importantly, most previous work in humans focused on young and middle-aged populations, and our results expand findings to an elderly population—suggesting that the biological processes that influence associations of Cer species with diabetes risk are relevant to older adults.

To date, most epidemiological studies that have assessed associations of sphingolipid species with incident diabetes in humans focus on Cer species, and few studies have assessed the impact of plasma or serum SM species on diabetes risk. Similar to the findings in this report, in the Strong Heart Study, SM-14, SM-16, SM-18, SM-20, SM-22, SM-24, HexCer-16, HexCer-22, HexCer-24, or LacCer-16 were not associated with diabetes risk (15). Likewise, the Latino Health Study reported that SM-16, SM-18, HexCer-16, and LacCer-16 are not associated with diabetes risk. However, findings from the Latino Health Study raised the possibility that SM-22 and SM-24 are associated with risk of diabetes; neither the findings reported herein nor our findings from the Strong Heart Study or Strong Heart Family Study confirmed those associations. However, we did not measure the fully saturated sphingomyelins (with a saturated fatty acid and a saturated sphingoid base) that were most strongly associated among the 32 SM species that were examined as part of the Latino Health Study (17).

Most published work in humans on Cers and sphingomyelins comprise observational studies to better understand associations of specific Cer and SM species with risk of chronic diseases, including type 2 diabetes (14, 15, 19). In order for these findings to be relevant in a clinical setting, more work is needed to assess ways to modify specific Cer and SM species and to test whether modifications in specific species influence health outcomes. Results of a small randomized trial/diet intervention called that SYSDIET Study suggest that consuming a healthy diet high in fruits and vegetables, whole grains, low-fat dairy products, lean meats, and vegetable oil reduced plasma levels of Cer-22 and Cer-24 over 12 weeks compared with a typical Nordic diet low in these foods (29). Moreover, results of secondary analyses of the SYSDIET Study suggest that the effect of plasma Cers on risk of CVD differs according to dietary pattern. In particular, in the SYSDIET Study, the effect of circulating Cers on risk of CVD was lower among participants randomized to a Mediterranean diet pattern enriched in nuts and olive oil compared with a traditional Western diet during a 1-year follow-up; this suggests that consuming a healthy diet may lessen the negative effects of Cers on CVD risk (30). Other known therapies include drug therapy; a 14-day regimen of 40 mg of simvastatin reduced Cers (i.e., Cer-16, Cer-18, Cer-20, and Cer-24) by approximately 25% in pre/post testing (31). Likewise, 5-week treatment with 10 or 40 g of rosuvastatin lowered total Cer levels by 33 and 37%, respectively, in men with the metabolic syndrome (32). Gastric bypass surgery among individuals with obesity has also been shown to lower levels of circulating Cers—and these effects were sustained for 6 months postoperatively (33). In addition, gastric bypass surgery has been shown to be associated with both diabetes prevention and remission in obese adults (34,35). Although most of these studies are small (n < 50)—results suggest that Cers may be modifiable by existing therapies, including diet change and drug intervention, and highlight the potential utility for measuring Cers in the clinical setting in the future. More (and larger) studies are needed to link these therapies (i.e., diet, drug therapy, gastric surgery) shown to influence circulating Cers in the short-term with long-term changes in circulating Cer levels and subsequent risk of diabetes in diverse populations, including both the general population and populations at high risk for diabetes. More research is also needed to better understand the influence of circulating sphingolipids on risk of diabetes-related complications in populations with prevalent diabetes, as well as associations of circulating sphingolipids with other chronic diseases, such as renal disease.

In exploratory analyses, additional adjustment for triglycerides attenuated associations of Cer-20 and Cer-22 with diabetes risk (but not Cer-16 or Cer-18 with diabetes risk). Comparing this finding to other studies is challenging since most previous studies in humans that have assessed associations of individual sphingolipid species with risk of diabetes did not adjust for triglycerides (14, 15, 17, 19) or did not contrast models unadjusted versus adjusted for triglycerides (16). However, in the Study of Latinos, a Cer risk score that comprised Cer-16, Cer-18, and Cer 42:3 was positively associated with diabetes risk in a model that did not include triglycerides; further adjustment for BMI, waist circumference, blood pressure, CRP, HDL, and triglycerides attenuated the finding. In addition, we have previously reported that plasma phospholipid arachidic, behenic, and lignoceric acids are inversely associated with diabetes risk in the CHS and 11 other cohorts that comprise the Fatty Acids and Outcomes Research Consortium—and that associations are attenuated after adjustment for triglycerides (36, 37). Attenuation of hazard ratios after adjustment for triglycerides may be explained at least in part because of triglycerides being a marker of insulin sensitivity and a sensitive marker of future diabetes risk (38).

This study has several strengths. CHS is a large prospective cohort study of risk factors for CVD in elderly adults in the United States. The sampling scheme (i.e., random sampling of eligible adults from Medicare eligibility lists) and the standardized methods for data collection employed by the CHS reduced the likelihood of selection and recall biases. Finally, the detailed data available on demographic, behavioral, and health factors maximized our capacity to control for potential confounders.

This study also has limitations. Although many sphingolipids were measured with good precision, it was not possible to examine the associations of all saturated fatty acids containing glycosyl-Cer species (e.g., HexCer-20, LacCer-20, LacCer-22, LacCer-24) because of analytical variability associated with the laboratory assay. In addition, we did not measure sphingolipids with unsaturated acylated fatty acids, and we only measured Cer and SM with the most common sphingoid base (d18:1). Frozen samples collected in 1992–1993 or 1994–1995 were used to measure sphingolipids; although it is unlikely that long-term storage of samples at −80 °C may have impacted sphingolipid concentrations, any alterations in sphingolipid composition because of storage would be expected to be nondifferential between future diabetes cases and noncases (39, 40, 41). In addition, we adjusted for several demographic, behavioral, and clinical factors that may be associated with sphingolipids and diabetes, but residual confounding by unmeasured or imprecisely measured factors is possible. In the CHS, plasma Cers and sphingomyelins were measured at one time point, and we are unable to assess if changes in plasma Cers and sphingomyelins over time are associated with risk of diabetes. Finally, as several of the Cer species of interest are correlated, it may not be possible to tease out the independent associations of each individual Cer species with diabetes risk.

## Conclusions

In conclusion, these results suggest that higher levels of circulating saturated fatty acids containing different Cer species (i.e., Cer-16, Cer-18, Cer-20, and Cer-22) are associated with a higher risk of diabetes in elderly adults. These findings support future efforts to explore pathways that explain how circulating Cers may influence risk of diabetes and to better understand the potential for lowering circulating Cers to be utilized as a novel target for diabetes prevention efforts.

## Data availability

The data that support the findings of this study are available from the CHS, but the study investigators are not allowed to distribute CHS data, and restrictions apply to the availability of these data. Requests for data are granted through the submission of a paper proposal or data repository request as outlined at: https://chs-nhlbi.org/CHS.

## Supplemental data

This article contains supplemental data.

## Conflict of interest

The authors declare that they have no conflicts of interest with the contents of this article.
