# Supplementary material for: Plasma ceramides containing saturated fatty acids are associated with risk of type 2 diabetes
Source: J Lipid Res. 2021 Sep 20;62:100119. doi: 10.1016/j.jlr.2021.100119 (PMC8517199; doi:10.1016/j.jlr.2021.100119)
Supplement: Supplemental Figs. S1–S3 and Table S1 [file mmc1.docx]

**ESM Figure 1. Baseline characteristics of study participants according to quartiles of ceramide species.** * denotes variables only measured at the exam in 1992-1993. The value of each covariate for the first and last quartile is provided in the figure (Q1, Q4). The values of each covariate across the 4 quartiles are also depicted with spark-lines. The colors of the spark-lines denote statistical significance of a test-for-trend based on a Bonferroni-corrected p-value of 0.05/19=0.0026 (based on 19 baseline characteristics of interest): Red, inverse association; grey, no association; blue, positive association. Abbreviations: BP, blood pressure; CHD, coronary heart disease; CRP, c-reactive protein; Cer-18, stearic acid containing ceramide; Cer-20, arachidic acid containing ceramide; Cer-24, lignoceric acid containing ceramide.

**ESM Figure 2. Baseline characteristics of study participants according to quartiles of sphingomyelin species.**  * denotes variables only measured at the exam in 1992-1993. The value of each covariate for the first and last quartile is provided in the figure (Q1, Q4). The values of each covariate across the 4 quartiles are also depicted with spark-lines. The colors of the spark-lines denote statistical significance of a test-for-trend based on a Bonferroni-corrected p-value of 0.05/19=0.0026 (based on 19 baseline characteristics of interest): Red, inverse association; grey, no association; blue, positive association. Abbreviations: BP, blood pressure; CHD, coronary heart disease; CRP, c-reactive protein; SM-14, myristic acid containing sphingomyelin; SM-18, stearic acid containing sphingomyelin; SM-20, arachidic acid containing sphingomyelin; SM-24, lignoceric acid containing sphingomyelin.


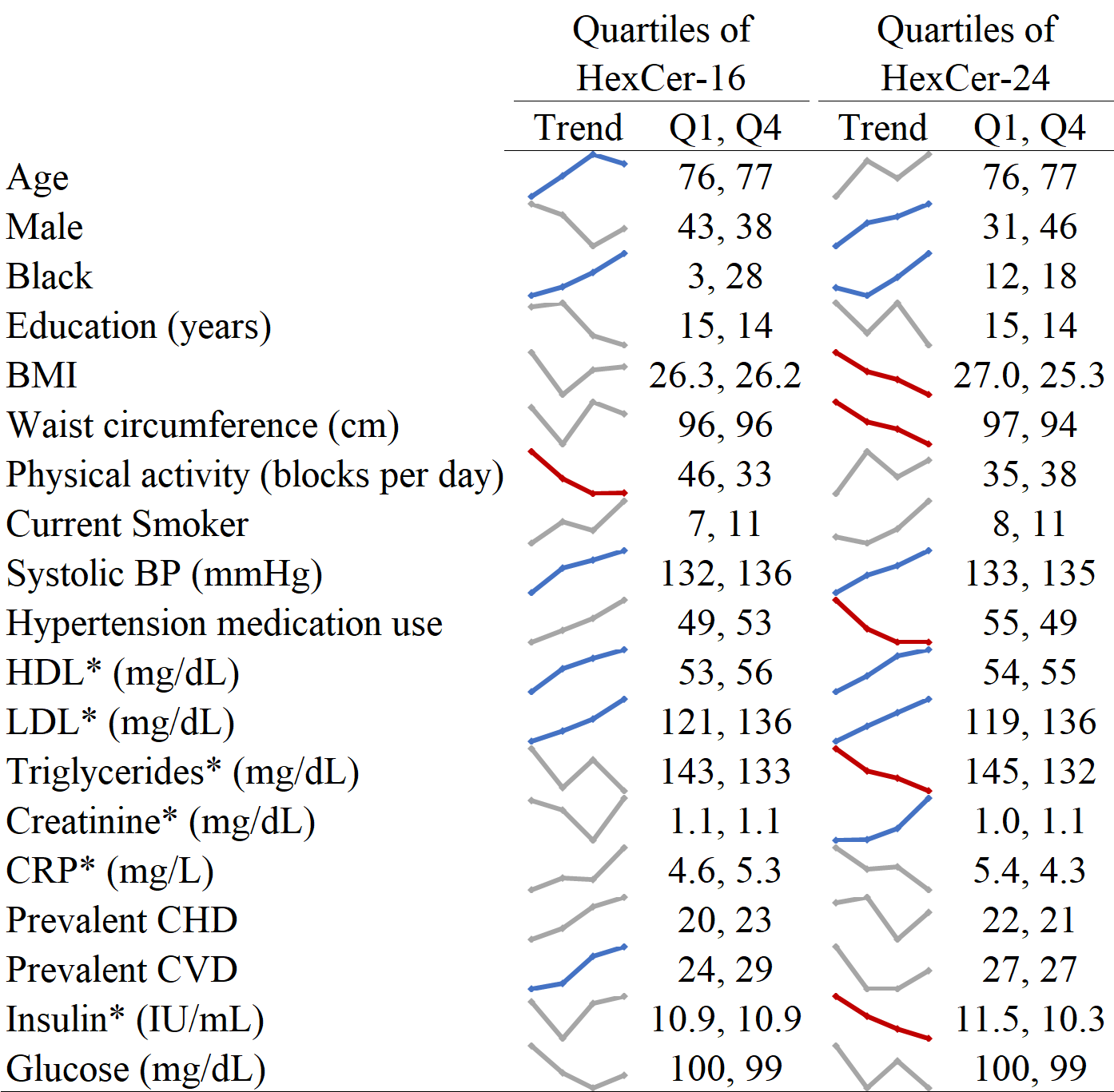


**ESM Figure 3. Baseline characteristics of study participants according to quartiles of glucosyl-ceramides and lactosyl-ceramide species.** * denotes variables only measured at the exam in 1992-1993. The value of each covariate for the first and last quartile is provided in the figure (Q1, Q4). The values of each covariate across the 4 quartiles are also depicted with spark-lines. The colors of the spark-lines denote statistical significance of a test-for-trend based on a Bonferroni-corrected p-value of 0.05/19=0.0026 (based on 19 baseline characteristics of interest): Red, inverse association; grey, no association; blue, positive association. Abbreviations: BP, blood pressure; CHD, coronary heart disease; CRP, c-reactive protein; HexCer-16, palmitic acid containing hexosyl-ceramide; HexCer-24, lignoceric acid containing hexosyl-ceramide.

| **ESM Table 1. Hazard Ratios (95% CI) for Incident Diabetes for Plasma Sphingolipids with Saturated Fatty Acids** | |
| --- | --- |
|  | **HR (95% CI)** |
| **Cer-16** | 1.13 (1.02, 1.26) |
| **Cer-18** | 1.14 (1.01, 1.27) |
| **Cer-20** | 1.03 (0.92, 1.15) |
| **Cer-22** | 1.04 (0.93, 1.17) |
| **Cer-24** | 0.97 (0.87, 1.09) |
| **SM-14** | 0.87 (0.78, 0.97) |
| **SM-16** | 0.99 (0.88, 1.11) |
| **SM-18** | 1.02 (0.91, 1.15) |
| **SM-20** | 0.95 (0.85, 1.05) |
| **SM-22** | 1.02 (0.91, 1.14) |
| **SM-24** | 1.01 (0.91, 1.13) |
| **HexCer-16** | 1.01 (0.91, 1.12) |
| **HexCer-22** | 0.94 (0.85, 1.04) |
| **HexCer-24** | 0.91 (0.82, 1.02) |
| **LacCer-16** | 0.97 (0.87, 1.07) |
| hazard ratios (95% CI) for diabetes per one standard deviation in log sphingolipid species concentration (µM). Adjusted for age, sex, race, enrollment site, education, smoking, physical activity, BMI, waist circumference, LDL cholesterol, triglycerides, year of sphingolipid measurement, and prevalent coronary heart disease. Abbreviations: HR, hazard ratio; Cer-16, palmitic acid containing ceramide; Cer-18, stearic acid containing ceramide; Cer-20, arachidic acid containing ceramide; Cer-22, behenic acid containing ceramide; Cer-24, lignoceric acid containing ceramide; SM-14, myristic acid containing sphingomyelin; SM-16, palmitic acid containing sphingomyelin; SM-18, stearic acid containing sphingomyelin; SM-20, arachidic acid containing sphingomyelin; SM-22, behenic acid containing sphingomyelin; SM-24, lignoceric acid containing sphingomyelin; HexCer-16, palmitic acid containing hexosyl-ceramide; HexCer-22, behenic acid containing hexosyl-ceramide; HexCer-24, lignoceric acid containing hexosyl-ceramide; LacCer-16, palmitic acid containing lactosyl ceramide. | |
